# Nature-Based Health Interventions for People with Mild to Moderate Anxiety, Depression, and/or Stress: Identifying Target Groups, Professionals, Mechanisms, and Outcomes Through a Delphi Study

**DOI:** 10.3390/ijerph23010126

**Published:** 2026-01-20

**Authors:** Louise S. Madsen, Knud Ryom, Liv J. Nielsen, Dorthe V. Poulsen, Nanna H. Jessen

**Affiliations:** 1 DEFACTUM, Central Denmark Region, 8000 Aarhus, Denmark; lmaden@rm.dk; 2 Department of Public Health, Applied Public Health, Aarhus University, 8000 Aarhus, Denmark; knudryom@ph.au.dk; 3 Research Unit for General Practice, Aarhus University, 8000 Aarhus, Denmark; livjuul1880@gmail.com; 4 Department of Geosciences and Natural Resource Management, University of Copenhagen, 1958 Frederiksberg, Denmark; dvp@ign.ku.dk

**Keywords:** nature-based health interventions, programme theory, mental health, anxiety, depression, stress, Delphi

## Abstract

**Highlights:**

**Public health relevance—How does this work relate to a public health issue?**

- Mild to moderate anxiety, depression, and/or stress are widespread and impair everyday functioning and quality of life.
- Current treatment options do not always meet the needs of this group, increasing interest in nature-based health interventions (NBHIs) in mental-health support.

**Public health significance—Why is this work of significance to public health?**

- The development of effective NBHIs requires a systematic, population-specific framework.
- This study identifies key elements; target group specifics, professional roles, mechanisms, and outcomes, essential for designing such interventions.

**Public health implications—What are the key implications or messages for practitioners, policy makers and/or researchers in public health?**

- NBHIs should be approached as complex interventions within a bio-psycho-social health perspective.
- The findings offer a shared foundation for practitioners, policy makers, and researchers to design and evaluate context-sensitive NBHIs.

**Abstract:**

Nature-based health interventions (NBHIs) are increasingly used in the healthcare system to support people with anxiety, depression and/or stress, highlighting the need for systematic development and evaluation. This study aims to identify target group, professionals, mechanisms, and outcomes of NBHIs for people with mild to moderate anxiety, depression, and/or stress. A Delphi-based study was conducted to explore core components of NBHIs in healthcare settings. Thirteen vs. eleven researchers with expertise related to the target group responded in two rounds. Respondents rated statements on a 7-point Likert scale and prioritised core components regarding target group, professionals, mechanisms, and outcomes. A thematic analysis was applied to synthesise qualitative responses. Consensus was achieved on 12 of 21 items across the four domains. Highest agreement concerned core mechanisms (nature interaction, social community, and physical activity), outcome priorities (mental wellbeing and quality of life), and professional competencies. Greater variation was observed regarding group composition and team delivery. Analysis of qualitative expert responses highlighted four key themes: (1) Balancing Group Composition, (2) Adapting Competencies to Context, (3) Core Mechanisms for Change, and (4) Weighing Perspectives in Outcome Selection. By setting out guiding principles for a programme theory, the study lays the foundation for the design and implementation of context-adapted NBHIs. The study underscores the need to approach NBHIs as complex interventions, thus contributing to a paradigm shift towards a new era of a bio-psycho-social health perspective.

## 1. Introduction

People with mild to moderate anxiety, depression, and/or stress experience complex and often long-term challenges that considerably interfere with daily functioning and diminish quality of life [1,2]. This presents a significant challenge as it places substantial demands on healthcare resources, often exceeding available capacity (due to healthcare utilization, labor market absenteeism, and socio-economic consequences in terms of healthcare costs and lost productivity) [3,4]. Additionally, existing treatment options may not be adequately tailored to meet the specific needs, e.g., regarding ‘difficult-to-treat depression’ in people who are unable to achieve an adequate therapeutic response despite pharmacotherapy [5], highlighting the necessity for more, sustainable, and scalable interventions.

In recent years, there has been increasing academic and societal attention on the positive impact of nature on mental health [6]. Research indicates that natural environments can reduce physiological stress responses, restore attentional capacity, and foster a sense of connectedness and meaning—particularly for individuals experiencing psychological distress [7,8,9,10,11,12]. Central to this development is the understanding that nature is not merely a passive backdrop but an active therapeutic agent that interacts with psychological processes. These central findings build on two distinct theoretical frameworks developed in the late 20th century: the Stress Reduction Theory, which draws on an evolutionary perspective to explain how natural environments elicit immediate affective responses such as perceived safety and reduced stress; and the Attention Restoration Theory which identifies four key environmental qualities: being away, extent, fascination, and compatibility, as essential for restoring depleted attentional capacities [13,14]. This recognition has led to a broader integration of nature-based health interventions (NBHIs) in public healthcare services, emphasising the importance of integrating green spaces and nature-based activities in programmes for mental health and well-being [15,16,17].

However, to develop relevant and sustainable context adapted NBHIs for people with mild to moderate anxiety, depression, and/or stress, there is a need for the design of a systematically developed framework that specifically addresses this target group [18,19]. Furthermore, interventions in natural environments are highly complex efforts, which need careful considerations both in the development phase, as well as in the evaluation. One way to work with such demanding interventions, is by using a complex intervention framework such as the Medical Research Counsil (MRC) framework [18], which highlight the need for a programme theory to develop and evaluate public health efforts. An often-central feat to programme theory is assessing mechanisms of change for interventions, which are often based on theory or practical knowledge. The MRC framework highlights the need to identify core aspects related to the target group (can we mix the target group), the professionals (which therapeutic competencies are necessary), and the underlying mechanisms (what are the active components) in NBHIs. Additionally, the MRC emphasises the selection of relevant measurement tools, as current assessments rely on a wide range of different outcomes (how do we measure the effect) [19].

*Nature Impact*, a national research project in Denmark, explores how NBHIs impact people experiencing mild to moderate anxiety, depression, and/or stress within a structured healthcare context [20]. *Nature Impact* seeks to inform the design of context adapted, evidence-based NBHIs that are feasible and can be effectively implemented into structured healthcare and therapeutic settings [20].

As part of this broader initiative, the present Delphi-based study aims to identify target group, professionals, mechanisms, and outcomes of NBHIs for people with mild to moderate anxiety, depression, and/or stress. The findings will inform the development of a programme theory adapted to the Danish healthcare context, forming the basis for the design and feasibility testing of context adapted NBHIs in Denmark.

## 2. Materials and Methods

### 2.1. Design

To gather structured insights and achieve reliable consensus on the target group, professionals, mechanisms and outcomes informing the development of design principles and implementation of NBHIs, a Delphi approach was selected [21,22]. The Delphi method is a systematic, iterative process, in which selected experts respond to multiple rounds of questions, with each round informed by anonymised feedback from the previous one [23]. To deepen understanding and nuance the interpretation of responses, open-ended questions were included alongside closed items.

A two-round modified Delphi technique was applied to address the study aim. The exploratory function of the first round in a traditional three-round Delphi survey, typically used to generate key issues, had already been fulfilled through preparatory work conducted as part of the *Nature Impact* research project, which provided a robust scientific foundation for the Delphi process. This made it possible to formulate the initial statements in advance, thereby streamlining the two Delphi rounds and ensuring a robust basis for expert evaluation.

In this study, “mild to moderate anxiety, depression, and/or stress” was used as a practice-based descriptor rather than a diagnostic category. Delphi respondents were asked to base their assessments on their professional experience with individuals who experience anxiety, depressive symptoms, and/or stress-related difficulties at a level typically addressed within municipal services, without the use of formal diagnostic criteria or standardised severity thresholds.

The target group was understood functionally as individuals showing signs of anxiety, depression, and/or stress where these difficulties constituted the primary cause of reduced daily functioning, thereby distinguishing actionable difficulties warranting intervention from transient or normative life stress.

### 2.2. Reflexivity Statement

The research team includes researchers with backgrounds in anthropology, medicine, public health, nursing, and physiotherapy. The primary qualitative analysis was conducted by an anthropologist, while clinical and practice-based perspectives were contributed by team members with experience in general practice, nursing, and physiotherapy. We recognise that these disciplinary positions may have influenced study design and interpretation, and reflexive discussions within the multidisciplinary team were used to critically examine assumptions and support analytical rigour.

### 2.3. Preparatory Work for the Delphi Development

**Figure 1:**
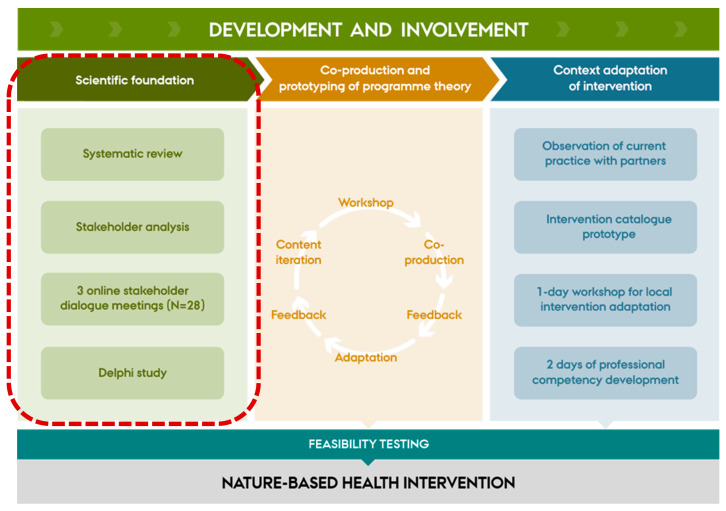
Illustration of the full development and involvement phases of the *Nature Impact* project informed by a co-production approach, including the scientific foundation forming the preparatory work of the Delphi process. The red dotted line box includes the scientific foundation behind the *Nature Impact* project.

The Delphi process was informed by a preparatory phase aimed at creating a strong conceptual and empirical basis for identifying essential content for developing the questionnaire items. This phase combined two overall complementary approaches: a systematic literature review investigating the effect of participation in NBHIs [19], and three online stakeholder dialogue meetings [24,25] with key stakeholders (*n* = 28). Together, these steps provided both evidence-based knowledge, knowledge gaps and practice-informed perspectives, forming part of the broader scientific foundation for the *Nature Impact* project (Figure 1).

The systematic literature review [19] examined the effects of NBHIs among people with mild to moderate anxiety and/or depression and individuals experiencing stress, with a particular focus on intervention design, target populations, and outcome measures. The review demonstrated that participation in NBHIs was associated with improvements in mental health outcomes, including reductions in anxiety, depression, and stress, as well as enhanced overall mental well-being. Across the 19 included studies, a total of 45 different outcome measures were identified, reflecting substantial heterogeneity in how intervention effects were evaluated. These measures were grouped into four overarching categories: symptom-specific scales, general mental health measures, nature- and therapy-related measures, and other health outcomes. The review further highlighted substantial knowledge gaps. In particular, there was limited empirical clarity regarding the mechanisms underpinning the therapeutic effects of NBHIs and how the therapeutic adjustment of activities may influence outcomes. These gaps directly motivated the stakeholder dialogue meetings and the Delphi process and informed the formulation of Delphi items, particularly those addressing target group composition and outcome selection (Appendix A).

Subsequently, three online stakeholder dialogue meetings were conducted to explore practical and experiential perspectives relevant to the development of NBHIs [25]. Participants (*N* = 28) were recruited through purposive sampling informed by stakeholder analysis and participatory mapping [26]. The sample included people with lived experience (*n* = 3), project practice partners (*n* = 8), general practitioners (*n* = 2), representatives from the Danish Nature Agency (*n* = 3), NGOs (*n* = 3), and private nature–health stakeholders (*n* = 9).

To situate the dialogues within the project’s developmental phase, preliminary findings from the systematic literature review [19] were presented to participants and used as an active point of departure for discussion. A semi-structured interview guide was applied, focusing on three overarching areas: (1) the relationship between nature and mental health; (2) organisational and structural conditions shaping the implementation of nature-based interventions; and (3) existing services, including barriers and opportunities related to reaching the target group and adapting interventions to participant needs. The discussions specifically addressed how existing evidence could be translated into practice and where experiential knowledge was needed to address gaps not covered by the literature.

The stakeholder dialogue meetings were analysed using thematic analysis [27,28]. Transcripts were coded independently by two researchers, followed by iterative discussions to refine and agree upon themes. The analysis aimed to synthesise shared perspectives, challenges, and practice-based considerations relevant to programme theory development.

Seven overarching themes were identified: (1) Needs of the target group, (2) Added value of nature, (3) Measuring the intervention, (4) Competency development, (5) Significance of the natural environment, (6) Structure and organisation, and (7) Societal potential. Guided by the aim of the Delphi study and its alignment with four core domains central to programme theory development within the MRC framework [18]:target group, professionals, mechanisms, and outcomes, themes 1–4 were retained to inform the formulation and structuring of the Delphi items. Themes 5–7, while analytically relevant, were excluded at this stage due to their broader contextual and systems-level focus. Traceability between stakeholder themes and specific Delphi items is provided in Appendix A.

### 2.4. Recruitment

Purposive sampling was used to invite experts with a minimum of five years of experience researching mental health, anxiety, depression and/or stress in Denmark to participate in the study. Expertise was defined as holding a research-related position (associate professor or professor) and having substantial research experience with the target population. To ensure consistency, we selected a homogeneous group representing only researchers, while aiming for variation in educational backgrounds and research fields, including public health, clinical psychology, general practice, psychiatry, rehabilitation, and mental health services. Our goal was to recruit 10–15 respondents. Seventeen researchers were invited via email informing about the project aim and methods and thus being able to respond to any questions prior to data generation and ensure that respondents could keep the planned timing. Of the total 17 researchers invited, 13 responded in the first Delphi round, and 11 continued their response into the second (Table 1). Respondents were leading researchers representing a broad range of professional backgrounds and academic disciplines (Table 1). While the Delphi panel comprised researchers only, the overall study design included practice-based stakeholders in both the preparatory phase and subsequent co-production of the programme theory (Figure 1).

### 2.5. Data Generation

Data was generated through two online survey rounds using the SurveyXact online platform (accessed in September-November 2024). Personalised links ensured that only respondents completing the first round were invited to the second round. Each round was open for 14 days, with a reminder email sent after seven days to non-respondents. Data collection took place from 25 September 2024 to 13 November 2024.

#### 2.5.1. Delphi Survey—Round 1

Prior to completing the survey, respondents (*N* = 13) provided informed consent for participation and data handling. The survey included 21 items divided into four domains related to target group composition, professionals, mechanisms, and outcomes for an evidence-based NBHI targeting people with mild to moderate anxiety, depression and/or stress (Appendix A). Example of an item was *“To what extent do you agree that nature-based health interventions should be delivered by an interdisciplinary team?”*. Respondents rated statements on a 7-point Likert scale (from “strongly disagree” to “strongly agree”).

To determine consensus in this study, a dual criterion was applied: first, a mean score of ≥ 5.0 was considered indicative of overall agreement; second, a solid consensus was more stringently defined as ≥ 70% of respondents selecting one of the three highest categories (5, 6, or 7), corresponding to “somewhat agree,” “agree,” or “completely agree.” It is important to notice that no uniform definition to when consensus is achieved exists [29]. When developing medical guidelines consensus is considered 75% [29] (however when including practice knowledge alongside research evidence (such as in this study), a lower % can be needed as consensus percentages. To provide more insight into our results, we have also highlighted a near consensus (60–69%) group, and a no consensus (<60%) group.

Additionally, a ranking task was included, asking respondents to prioritise the following outcomes: symptoms, quality of life, mental well-being, and functioning. Each respondent assigned a unique rank order (1 = most relevant), enabling identification of relative outcome priorities across the group. Based on insights from the preparatory phase, including findings from the systematic literature review and stakeholder dialogue meetings, nature connectedness was identified as a theoretically central outcome. Because of this strong conceptual grounding, it was pre-selected prior to the Delphi rounds and therefore excluded from the ranking exercise.

Each thematic section ended with an open-ended text field, allowing respondents to elaborate and provide additional input. For instance, respondents could suggest additional mechanisms; suggestions mentioned by more than one respondent were reviewed and, if relevant, included in the second round.

#### 2.5.2. Delphi Survey—Round 2

Following the first round, the research team summarised and synthetised all responses and comments, revising the second-round questionnaire for improved clarity and precision based on respondent feedback. Therefore, in round two, we adopted a more exploratory approach, encouraging respondents (*N* = 11) to elaborate on their responses from round one (Appendix B). The aim was to collect more nuanced and detailed feedback. Results from the first round were presented visually using bar charts to aid interpretation. Selected domains were included for further prioritisation in the second round, while items that had already reached consensus were presented for information only and not reassessed. In response to comments in Round 1, an open-ended question was added in Round 2 to examine possible adverse effects. As in the first round, respondents were invited to elaborate on their responses through open-text comment fields linked to each question.

### 2.6. Analysis

#### Delphi Survey—Round 1 and 2

The analysis was conducted in two sequential and complementary phases. First, a descriptive analysis of the quantitative data from both Delphi rounds was performed to identify levels of consensus and patterns of prioritisation across items. These results informed the subsequent qualitative analysis by highlighting areas of agreement, divergence, and uncertainty requiring further interpretation.

Second, the open-ended responses were analysed using Thematic Analysis as described by Braun and Clarke [27,28]. Initial coding was conducted by the first author, who led the qualitative analysis. The primary analyst’s anthropological training supported sensitivity to context, meaning-making, and interpretive nuance, while clinical and practice-based perspectives contributed to grounding the analysis in healthcare realities.

Codes were developed abductively as the four predefined domains of the Delphi study: target group, professionals, mechanisms and outcomes, provided an overarching analytic frame. Data within each domain was analysed inductively. Coding and emerging interpretations were discussed iteratively within the multidisciplinary research team, allowing for critical reflection, refinement of codes, and the development of themes. Analytical rigour was supported through reflexive dialogue, where assumptions, alternative interpretations, and disciplinary perspectives were actively examined.

Themes were developed through an iterative process of refinement and abstraction and were structured into a narrative that both reflected and elaborated upon the quantitative findings. Selected illustrative quotes are presented in the Results section to support transparency and demonstrate how themes were grounded in the data.

## 3. Results

Across both rounds, respondents evaluated and prioritised questions related to group composition, team formats and competencies, mechanisms of change and outcome selection. The quantitative part of the Delphi process offered insights into where expert consensus existed, and where divergence remains, on key considerations for developing NBHIs for people with mild to moderate anxiety, depression, and/or stress. Table 2 illustrates how consensus was reached in 12 out of 21 items in total, with 9 items reflecting divergence in the field.

No consensus was reached regarding the optimal group composition for NBHIs. When asking respondents whether people with mild to moderate anxiety, depression, and/or stress benefit most from diagnosis-specific groups, the mean score was 4.1. For mixed- groups, the mean score was slightly higher at 4.4. Only 46% of respondents agreed (5–7) on each statement, indicating divergent expert opinions.

Respondents were asked whether NBHIs should be delivered by an interdisciplinary team. While the item did not formally meet the predefined threshold for consensus (≥70% agreement in categories 5–7), responses strongly indicated support for interdisciplinary delivery of NBHIs. The mean score was 5.6, and 69% of respondents expressed agreement, placing it just below the consensus cut-off. Respondents expressed a clear preference for including health-related and nature-specific competencies in the delivery of NBHIs. In relation to identifying relevant professional backgrounds, physiotherapists reached full consensus with 100% agreement, followed by nature educators (84%) and psychologists (77%), all exceeding the predefined consensus threshold. While nurses (68%), medical doctors (69%), and occupational therapists (61%) did not reach formal consensus, their inclusion still received moderate support.

There was strong consensus that professional expertise is essential in delivering NBHIs, but respondents clearly distinguished between types of knowledge. The highest agreement was found for professionals’ knowledge and experience with the target group: people with mild to moderate stress, anxiety, and/or depression (mean 6.5; 92% agreement). Slightly lower, though still at consensus level, was the emphasis on knowledge of NBHIs themselves (mean 6.3; 77% agreement).

Regarding mechanisms of change necessary for NBHIs, respondents reached consensus on seven out of ten proposed items. The most highly endorsed mechanisms were participation in everyday life (mean 6.3; 100% agreement), social communities (6.6: 92% agreement), and nature experiences and interaction (6.3; 84% agreement). Based on respondents’ feedback in Round 1, mindfulness/presence was added as an additional mechanism and included in the prioritisation exercise in Round 2. Respondents were asked to rank the mechanisms in order of perceived relevance (1 = most relevant) as illustrated in Table 3.

This approach produced a clear hierarchy: when asked to prioritise mechanisms directly against one another, respondents appeared to reconsider their initial ratings. In contrast to Round 1, where participation in everyday life received the highest level of consensus (100%), it was ranked lowest in Round 2. While participation in everyday life remains broadly endorsed as a desirable outcome, it was perceived as less central to the mechanisms of change in NBHIs than more immediate processes such as nature interaction and social communities. The most highly prioritised mechanisms in Round 2 were nature experiences and interaction, social communities, and physical activity, all of which had reached consensus in the first round.

Finally, respondents were asked to prioritise relevant outcomes for evaluating NBHIs. As this approach was only to gather inputs not consensus, a clear pattern of prioritisation emerged (1 = most relevant) (Table 4).

The table shows the outcomes ranked by respondents in order of perceived relevance. Lower mean scores indicate higher prioritisation.

Respondents were asked to rank four outcome domains in order of importance for evaluating NBHIs (1 = most important). Mental well-being was ranked highest (mean 1.4), followed by quality of life (2.7), functioning (2.8), and symptom reduction (3.2). Although the exercise did not seek to establish consensus, the resulting order indicates shared preferences regarding outcome focus on the evaluation of NBHIs in Denmark.

Together, these findings provide a structured overview of where expert agreement converges and where complexity remains. The results offer a quantitative foundation for the more nuanced, qualitative perspectives explored in the following four themes.

### 3.1. Key Insights Through Open-Ended Questions

Additional to the consensus seeking questionnaire, the open-ended questions generated rich insights into key considerations for designing and evaluating NBHIs targeting people with mild to moderate anxiety, depression, and/or stress, and four main themes were identified: Balancing Group Composition, Adapting Competencies to Context, Core Mechanisms for Change, and Weighing Perspectives in Outcome Selection. Together, these themes reflect areas of both consensus and nuanced deliberation, providing a foundation for the development of evidence-informed, context-adapted NBHIs.

#### 3.1.1. Balancing Group Composition

While the quantitative findings showed no clear consensus on group composition, respondents’ qualitative responses shed light on underlying reasoning. A modest preference for mixed groups was explained through recognition of shared experiences, high comorbidity, and symptom overlap between anxiety, depression, and stress—factors that challenge rigid diagnostic separation.

*“I consider mixed groups to be advantageous, partly because individual diagnoses or conditions often overlap or co-occur, and partly because participants are likely to recognise key challenges across conditions, even when they are categorised differently. Moreover, most—if not all—psychiatric conditions are closely linked to levels of stress.”*

Mixed groups were described as more reflective of daily practice complexity and as enabling peer mirroring, emotional validation and feelings of normalisation.

Several respondents noted that the natural environment itself may facilitate a transdiagnostic process, where diagnostic labels become less salient in favour of shared experiences such as calm and connection. Mixed group formats were also seen to support broader personal development, whereas diagnosis-specific groups could risk reinforcing problem-focused identities.

However, respondents emphasised that the optimal group composition should align with the intervention’s structure and target group. For individuals with severe symptoms or where diagnosis-specific psychoeducation is central, targeted groups may be more appropriate. In the absence of definitive evidence favouring one model, practical factors, such as group size and resources, were also cited as important.

#### 3.1.2. Adapting Competencies to Context

Although respondents generally supported the idea of interdisciplinary teams in NBHIs, views also reflected variation and context sensitivity. Qualitative insights underscored that team structure should remain flexible and shaped by the intervention’s goals, target group, and how nature is integrated. Rather than prescribing fixed professional roles, respondents highlighted the importance of combining psychological, physical, and nature-based expertise, adapted to the specific focus and setting of the intervention.

Many noted that physical activity, sensory engagement or psychoeducation require different professional profiles, and that team structure should reflect what is needed for the context adapted NBHI. While physiotherapists and psychologists were preferred, professional roles like nurses and medical doctors were seen as important contributors in planning and consultation, helping to integrate NBHIs within the broader healthcare system. Respondents also emphasised that working in nature can benefit practitioners themselves, potentially enhancing job satisfaction and sustainability. Calls to broaden the definition of NBHIs included suggestions to involve less conventional professionals, such as anthropologists or philosophers, to support existential and holistic perspectives.

There was strong agreement that professionals should possess both knowledge of NBHIs and insight into the target group.

*“The ideal facilitators for running these groups are, in my view, individuals who understand and can recognise mental illness or psychological vulnerability, while at the same time being able to introduce and work with nature in a credible way. This provides an equal sense of safety and authenticity.”*

Respondents underlined the need for professionals who can sensitively navigate psychological vulnerability while engaging with nature in a credible way. The depth of nature-related competencies required depends on whether nature is used as a setting or an active therapeutic tool.

#### 3.1.3. Core Mechanisms for Change

Respondents described nature experience and interaction, social community participation, and physical activity as interrelated processes central to NBHIs. These mechanisms reflect the embodied and relational dimensions of NBHIs and were seen as foundational for enhancing therapeutic outcomes, particularly by fostering connection, safety and movement.

*“There is considerable overlap between several of the mechanisms, and I fundamentally believe that they are deeply intertwined and mutually conditioning. However, without an invitation into a group, without movement (physical activity), and without interaction with nature, I do not think it is possible to achieve the other effects or outcomes, which I would consider more or less secondary.”*

At the same time, respondents stressed that the mechanisms do not operate in isolation. Many were described as overlapping or mutually reinforcing, such as empowerment and self-efficacy, or nature interaction and sensory engagement, making strict ranking inadequate to capture their interdependence. The ranking approach was therefore seen less as a fixed hierarchy and more as a reflective dialogue about emphasis, context and sequencing.

Respondents also pointed to the importance of individual variability. Mechanisms such as body awareness or mindfulness, while described as beneficial for some, were reported by several participants as potentially distressing for individuals who struggle with heightened internal stress or trauma-related reactions. Respondents noted that these practices may increase awareness of internal sensations in ways that some participants find overwhelming, underscoring the need for careful tailoring. Several respondents called for greater attention to unintended effects and “competing mediators,” urging a shift from asking what works? to what works for whom, and under what conditions? Risks such as emotional overload, accessibility barriers, or post-programme isolation were viewed not as flaws in NBHIs, but as context-dependent outcomes requiring careful consideration.

In summary, respondents broadly agreed that NBHIs should actively incorporate nature interaction, social communities, and physical activity as core therapeutic mechanisms. These were seen as central not only due to their inherent value, but also because they reflect what NBHIs can uniquely offer. At the same time, respondents emphasised that mechanisms must be matched to the needs, capacities, and preferences of individuals. Rather than prescribing fixed components, interventions should be designed with professional sensitivity, allowing for flexibility, responsiveness, and attention to potential unintended effects.

#### 3.1.4. Weighing Perspectives in Outcome Selection

The selection of outcomes for evaluating NBHIs generated both agreement and thoughtful divergence. Respondents ranked mental well-being as the most relevant outcome, with symptom reduction ranked lowest. Thus, Round 1 reflected a shared preference for positive, rehabilitation-oriented measures over narrower, clinical symptom metrics. However, in Round 2, when asked to reflect more deeply on the choice between everyday functioning and symptoms, more nuanced perspectives emerged.

*I fully recognise and agree upon the dilemma: functioning seems more relevant and timely, whereas symptoms are more established and institutionally recognised.*

Many respondents viewed everyday functioning as a more holistic and person-centred outcome, particularly relevant in rehabilitation or preventive contexts. It was seen to better reflect how people manage and live with their symptoms, rather than simply tracking their presence. Conversely, others emphasised the continued relevance of symptom measures, especially for demonstrating clinical effectiveness, enabling comparability with other studies, and meeting research and policy standards. While not the most person-centred, symptoms were viewed as strategically important for gaining recognition within the structured health care system. Patient Reported Outcome Measures were highlighted as a possible bridge between subjective experience and standardised metrics. Respondents broadly supported a focus on well-being, everyday functioning, and quality of life, while also acknowledging the strategic value of symptom tracking in implementation and research.

## 4. Discussion

This study identified key elements for developing effective NBHIs for people with mild to moderate anxiety, depression, and/or stress, including core mechanisms, outcomes, and required professional competencies. While consensus was achieved on these elements, variation in group composition and delivery underscored the need for flexible, context-sensitive approaches and outcomes capturing both holistic wellbeing and clinically meaningful change.

This need for flexibility is consistent with a growing body of literature describing the field as fragmented and lacking in conceptual coherence. As White and colleagues argue, the current proliferation of terms, practices, and theoretical traditions creates challenges for the cumulative development and evaluation of NBHIs [30]. A recent systematic review of reviews identified thirteen overlapping categories within the field, including forest therapy, wilderness-based interventions, care farming, blue space interventions, nature play, horticulture, and green exercise, each with its own conceptual lineage, setting, and health logic [31]. This breadth reflects the field’s richness but also presents a challenge when developing practice, evaluation, and policy implementation frameworks. NBHIs are inherently complex and diverse, and full standardisation is neither possible nor desirable [18,32].

Building on this premise, the present Delphi study contributes to shaping a flexible and integrative structure. Rather than prescribing fixed formats, it identifies central considerations across four interrelated domains: the target group participating in NBHIs, the professionals delivering them, the mechanisms of change involved in NBHIs, and the outcomes used to evaluate impact. These domains do not constitute a rigid model but offer a spectrum of best-practice principles to support local adaptation while maintaining theoretical and practical coherence. The following discussion examines each domain in turn and contextualises the findings within the broader literature.

### 4.1. Discussing the Target Group, Professionals, Mechanisms, and Outcomes

Complex interventions like NBHIs developed for the benefit of the target group, begin with the people involved and the considerations surrounding group composition. In this study, respondents generally supported mixed groups, arguing that anxiety, depression and/or stress are related conditions and thus respondents may benefit from shared processes of reflection, activity and connection. This perspective aligns with recent evidence highlighting the high comorbidity and diagnostic fluidity between these conditions, particularly in primary care [33]. While stress is not a clinical diagnosis per se, it often co-occurs with subclinical or diagnosed anxiety and depression, creating blurred clinical boundaries that challenge categorical approaches [34]. These conditions are also dynamically interrelated: severe stress may exacerbate anxiety and depressive symptoms, while untreated anxiety or depression can increase stress levels.

At the same time, open-ended responses acknowledged that diagnosis-specific groups may be appropriate in certain contexts, such as when addressing acute psychological vulnerability, trauma, or distinct therapeutic requirements. This reinforces the importance of clarifying the intended target group and symptom severity [31,34]. For NBHIs employing a mixed group format, respondents should be experiencing mild to moderate levels of anxiety, depression and/or stress, with more severe or acute conditions considered a contraindication. This approach reflects a broader shift in mental health and rehabilitation toward need-based, context-sensitive practices rather than strict diagnostic segmentation [35].

The diversity of mental health experiences not only calls for careful consideration of who participates in NBHIs but also informs the composition of professional teams and the competencies required for effective delivery. Findings from this study suggest that NBHIs are best supported through interdisciplinary collaboration, bringing together diverse professional perspectives across disciplines during both planning and implementation. This underscores the need for health professionals who not only draw on their disciplinary expertise but also navigate psychological, physical, and environmental dimensions of health [36].

The findings of this study suggest a prioritisation of psychological, physical, and nature-based competencies in NBHI delivery. Population-specific insight was regarded as the most critical foundation, with nature experience and intervention knowledge seen as essential in combination. This points to the need for professionals who can integrate a deep understanding of the mental health context of intervention participants with specialised knowledge of nature-based intervention approaches. As described by Bloomfield [37], a facilitator of NBHIs should be able to speak (at least) two different ‘languages’: the language of healthcare and practice, and the language of nature and environmental engagement. When nature is not merely the setting but the active medium of change, this dual fluency becomes essential, as facilitators must possess the capacity to translate nature experiences into therapeutic value [38,39].

In development, promotion and delivery of effective NBHIs health professionals play a key role [39]. However, a lack of training and confidence in delivering interventions outside conventional clinical settings remains a recognised barrier, particularly regarding the targeted and therapeutic use of natural environments [32,40,41,42]. This underscores the need for strategic investment in upskilling, ensuring that the competencies required to deliver NBHIs are not assumed but actively cultivated through education, guidelines, and organisational support, as also concluded by Stanhope and colleagues [43].

Ultimately, the relevance of professional competencies cannot be separated from the mechanisms that drive change in NBHIs. Identifying the factors that influence the effectiveness of these interventions in promoting health and wellbeing remains a key research need [41]. In this Delphi study, three core mechanisms of change reached consensus and were ranked as most important: (1) nature experience and interaction, (2) social community, and (3) physical activity. Together, these mechanisms reflect an embodied and relational understanding of how NBHIs support mental health. This multi-faceted approach distinguishes NBHIs from treatment as usual for individuals with mild to moderate anxiety, depression, and/or stress, where interventions are typically pharmacological or non-pharmacological treatments others than NBHIs, especially regarding depression [44]. By contrast, NBHIs integrate sensory, social, and physical dimensions of experience, aligning therapeutic processes with the environments in which people live and recover.

However, the marked drop in the prioritisation of participation in everyday life between Round 1 and Round 2 requires careful interpretation. One explanation is that the shift reflects a distinction between what respondents value in principle and what they consider a primary therapeutic driver. A further explanation, raised by several respondents, concerns conceptual ambiguity: elements such as participation in everyday life may be understood more as outcomes than as mechanisms of change. When required to weigh mechanisms directly against one another, respondents therefore prioritised processes more closely linked to immediate therapeutic action, nature interaction, social connection, and physical activity. Another plausible interpretation is that the change does not represent a shift in expert opinion but instead stems from differences between the rating and ranking formats. Likert-scale ratings allow multiple mechanisms to be endorsed as important simultaneously, whereas a forced-ranking task requires respondents to differentiate between them, even when they are conceptually overlapping. In this sense, the ranking exercise may have revealed relative prioritisation rather than altered underlying views.

The three mechanisms identified in this study also correspond with three of the eleven key moderating and mediating factors influencing the effectiveness of NBIs for overall health and wellbeing, as identified by Kaleta and colleagues [31]. Each of these mechanisms is well supported in existing research, offering valuable insights into their individual contributions to health and wellbeing. Interacting and connecting with nature has, over the past decade, developed into an established and impactful research field, with a growing body of consistent evidence demonstrating its effectiveness as a pathway for promoting mental wellbeing [17,19,41,45,46,47]. A related yet distinct line of evidence concerns the role of physical activity. Nature not only facilitates engagement in movement but can also enhance both its amount and intensity, which is why activity is an inherent element of many NBHIs [48,49]. In particular, green exercise interventions have been shown to effectively enhance mental wellbeing [50]. The social dimension of NBHIs is likewise highlighted in both this Delphi study and the wider literature, where group-based settings are found to enhance outcomes [31,37]. Positive social communities and supportive interactions within NBHIs may reduce hopelessness and improve self-worth among people experiencing depression [49].

A key contribution of this Delphi study is the recognition that the identified mechanisms of change: nature interaction, social community participation, and physical activity, operate not as discrete components but as interdependent processes that mutually reinforce one another. This aligns with for instance, the theoretical foundations of the Stress Reduction Theory posits that natural environments elicit rapid affective and physiological downregulation, which may in turn enhance individuals’ capacity to engage socially and participate meaningfully in group activities [13]. Similarly, the Attention Restoration Theory suggests that environmental features such as fascination and being away replenish cognitive resources, potentially enabling deeper social connection and facilitating motivation for physical engagement [14]. These key mechanisms may therefore function synergistically: affective calming through nature interaction may create the conditions for social safety; cognitive restoration may support reflective dialogue; and physical activity may strengthen both mood regulation and connectedness to place.

Taken together, the findings underscore the need to conceptualise NBHI mechanisms within a bio-psycho-social paradigm, in which body, environment, and social processes are entangled rather than separable. Rather than viewing mechanisms as linear or sequential, NBHIs may derive their therapeutic potential precisely from the ways these processes co-occur and amplify one another. This reinforces the importance of designing interventions where nature serves not simply as a setting but as a primary therapeutic agent shaping psychological, social, and behavioural change [31].

While the core mechanisms describe how change is facilitated, the outcomes reflect what that change aims to achieve. The study findings reveal a constructive tension between person-centred relevance and clinical legitimacy, underscoring the need to weigh more perspectives in outcome selection. In the field of NBHIs, there is no single or definitive answer as to which outcomes should be prioritised [31,41,51]. A recent review by Jessen and colleagues on the effects of NBHIs for individuals with anxiety, depression and/or stress identified 45 distinct outcome measures, illustrating the diverse ways in which contact with nature influences mental health and wellbeing [19]. This Delphi study contributes by clarifying key considerations for selecting and framing outcomes in this context.

The findings reveal both convergence and constructive divergence in respondents’ views on relevant outcomes for NBHIs. A strong preference for mental well-being over symptom reduction reflects a broader shift toward positive, rehabilitation and recovery-oriented outcomes [35]. At the same time, the debate around everyday functioning versus symptom improvement illustrates the layered complexity of outcome selection [52]. This duality underscores the ongoing tension between clinically legitimised metrics and outcomes that resonate with lived experience. Patient-reported outcome measures were identified as a potential bridge between these domains, combining subjective perspectives with the rigour of standardised assessment.

At a broader level, this tension illustrates the challenges of translating interventions from a biomedical framework into bio-psycho-social practice settings [18,53]. The biomedical tradition in mental health research has historically privileged symptom reduction as the primary indicator of success [53]. However, when interventions are situated in outdoor, relational, and dynamic environments, such measures become increasingly insufficient [18]. Respondents implicitly recognised this shift, often emphasising outcomes related to well-being, quality of life, and everyday functioning, dimensions more consistent with a bio-psycho-social orientation. Notably, even those who advocated for maintaining symptom measures did so not in opposition to holistic outcomes, but in acknowledgment of the institutional structures that continue to privilege biomedical legitimacy [53].

Collectively, the discussions of target group, professionals, mechanisms, and outcomes in NBHIs highlight a field in transition, moving toward more integrative and context-sensitive understandings of intervention design [54]. This momentum naturally connects to broader developments in health research methodology, particularly the increasing recognition of complexity in intervention science [18].

### 4.2. Towards a New Era in Nature-Based Health Interventions

There is a growing recognition of the necessity to conceptualise public health interventions as complex interventions, as articulated in the 2021 update of the MRC framework [18]. This approach acknowledges the complexity of such interventions, which involve interacting components, dynamic contexts, and non-linear causality. NBHIs is an example of such interventions operating within diverse systems and targeting multiple outcomes, challenging the linear and standardized assumptions of traditional biomedical models.

The MRC framework provides a valuable lens through which to address these complexities inherent to the NBHIs, encouraging researchers and practitioners to consider not only what works, but how, for whom, under what circumstances, and why. By framing NBHIs as complex, the MRC framework highlights the importance of theory-informed design, iterative development through co-production and stakeholder engagement, and contextual sensitivity. It also emphasises the need for mixed-methods/multimethod approaches that capture both measurable outcomes and the nuanced processes through which change occurs [18]. This is particularly relevant in nature-based settings, where individual experiences of nature, the role of facilitators, group dynamics, seasonal and environmental variability, and place-based meanings all contribute to the intervention’s effects [55]. Furthermore, this perspective acknowledges the challenges of implementation and scalability. NBHIs often rely on cross-sector collaboration and are shaped by local resources, values, and infrastructures [56]. Thereby, findings from this Delphi-based study support the assumption that the MRC framework is particularly well-suited for guiding the development and evaluation of NBHIs. Recognising and acknowledging NBHIs as complex interventions highlights the need for new approaches to their development and evaluation—marking a shift towards a new era in NBHIs [54].

### 4.3. Methodological Considerations

This study applied a modified two-round Delphi design [21,22], preceded by a preparatory phase consisting of a systematic literature review and stakeholder dialogue meetings [25]. This approach strengthened content validity by grounding the questionnaire in both empirical evidence and practice-based insights [57]. The inclusion of open-ended questions alongside Likert-scaled items enabled both structured prioritisation and deeper interpretive input, crucial in a field characterised by interdisciplinary perspectives and conceptual ambiguity [21].

The combined use of descriptive statistics and Reflexive Thematic Analysis may be viewed as unconventional from a traditional Delphi perspective. However, this integrative approach was chosen to balance numerical consensus with interpretive depth, aligning with the complex and interdisciplinary nature of NBHIs, and reflected in the richness and nuance of respondents’ responses.

The expert panel was relatively homogeneous, comprising senior mental health researchers with limited direct experience in nature-based practices [21]. Although the Delphi panel was limited to researchers, practice-based stakeholders were involved in the preparatory phase and are re-engaged after the Delphi rounds to co-produce the final programme theory (Figure 1). This staged approach separates conceptual consensus-building from implementation refinement, but delivery-related findings should be interpreted with this limitation in mind.

Further the panel composition likely shaped the pattern of findings [58]. The panel’s strong theoretical and clinical orientation may explain the relatively high consensus on conceptual and mechanism-related items, while contributing to greater variation in delivery-oriented items, including group composition and team structure. This focused expertise enhanced internal consistency but may have narrowed the range of perspectives on nature-specific competencies and mechanisms, limiting transferability to broader practice settings. These aspects typically rely on experiential or practice-based insights that were underrepresented in the panel. Consequently, findings related to mechanisms and outcomes can be considered theoretically robust and well-grounded in existing mental health evidence. In contrast conclusions concerning delivery formats and professional competencies would benefit from further validation through engagement with practitioners and people representing the target group.

Attrition between Round 1 and Round 2 was limited to two respondents. A comparison of their Round 1 responses with those of respondents who completed both rounds indicated similar patterns of ratings and qualitative inputs, suggesting that attrition was unlikely to have had a substantive impact on the results.

Another limitation relates to the preparatory phase, where stakeholder consultations did not include representatives from voluntary or community-led nature-based organisations. While this decision ensured alignment with the formal healthcare context in which the intervention will be implemented, it may also have biased the conceptual framing toward professionalised models of NBHIs. As voluntary-sector providers often operate with different logics, values, and practices, their absence may have constrained the diversity of perspectives that informed the early design assumptions.

With benefits to both human well-being and pro-nature conservation behaviors, nature connectedness is emerging as an important psychological construct for a sustainable future [10]. In this study, the selection of a nature connection outcome scale was decided prior to the Delphi process to ensure theoretical alignment [31,59]. We acknowledge that the decision to pre-select nature connectedness as an outcome, although theoretically justified, introduced a structural bias into the Delphi process. By defining one outcome as central before expert consultation, the ranking of remaining outcomes may have been influenced, limiting the extent to which outcome prioritisation reflected unrestricted expert deliberation.

The lack of formal diagnostic or screening-based operationalisation of symptom severity should be considered a limitation of the study. However, this reflects the practice-based context of municipal service provision, where eligibility and intervention needs are typically assessed in functional rather than diagnostic terms.

Overall, the study ensured internal coherence within a clinical research context but may have constrained the inclusion of applied, practice-based perspectives. The findings are thus most transferable to structured healthcare settings and should be interpreted within this disciplinary context [58].

## 5. Conclusions

By synthesising expert perspectives through a structured Delphi process, the study provides a comprehensive overview of the elements that should inform intervention design, delivery, and evaluation of NBHIs in people with mild to moderate anxiety, depression and/or stress. These insights contribute to the ongoing advancement of evidence-based practice by bridging conceptual understanding with practical guidance for healthcare professionals, researchers, and policymakers. Furthermore, the study highlights the complexity inherent in the development and evaluation of NBHIs, underscoring the importance of approaching these as complex interventions. By embracing this complexity, rather than seeking to reduce it, NBHIs can be understood and assessed within a broader bio-psycho-social framework. This perspective not only reflects the multifaceted nature of human–nature interactions but also signifies a potential shift from a predominantly biomedical paradigm towards a more integrative understanding of mental health and wellbeing.

The findings establish guiding principles that can inform the development of a robust programme theory for NBHIs. Such a framework not only clarifies how and why these interventions may work but also facilitates the adaptation of NBHIs to diverse contexts within the Danish healthcare system. By laying a foundation for context adaptive design and implementation, this study supports the integration of NBHIs into mental health care pathways and underscores their potential as complementary approaches to conventional treatments. Future research should build upon these principles to test and refine intervention models, evaluate their effectiveness in real-world settings, and explore long-term impacts on healthcare delivery and patient outcomes.

## Figures and Tables

**Table 1:** Sociodemographic of respondents Round 1 and 2.

Variable	Round One		Round Two	
	N	%	N	%
**Gender**				
Male	7	54	6	55
Female	4	31	3	27
Other/prefer not to answer	2	15	2	18
**Position Title**				
Professor	8	61	6	55
Associate Professor	5	39	5	45
**Professional Background**				
Doctor	4	31	4	37
Physiotherapist	2	15	1	9
Psychologist	3	23	3	27
Sports Science	1	8	1	9
Mental Health	3	23	2	18
**Primary Research Field**				
Public Health	2	15	1	9
Clinical Psychology	1	8	1	9
General Medicine	1	8	1	9
Psychiatry	2	15	2	19
Rehabilitation	4	31	3	27
Mental Health	3	23	3	27

**Table 2:** Expert agreement on key aspects of NBHIs (first Delphi round).

Rank	Item	Mean	% Agreement (5–7)	Consensus Cut-Off
	*Domain 1. Target group:* *how participants in NBHIs should be grouped*			
1	Mixed-diagnostic group intervention	4.4	46%	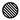
2	Diagnosis-specific group intervention	4.1	46%	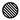
	*Domain 2.a. Interdisciplinary delivery:* *whether NBHIs require an interdisciplinary team*			
1	Delivered by interdisciplinary team	5.6	69%	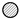
	*Domain 2.b. Professional groups involved:* *which professional backgrounds should deliver NBHIs*			
1	Physiotherapist	6.2	100%	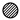
2	Nature Educator	5.7	84%	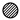
3	Psychologist	6.2	77%	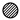
4	Nurse	4.9	69%	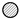
5	Medical doctor	5.0	68%	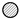
6	Occupational Therapist	5.8	61%	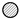
	*Domain 2.c. Core competencies of professionals:* *core competencies for NBHI planning and delivery*			
1	Experience with the target group	6.5	100%	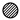
2	Experience with NBHIs	6.3	100%	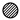
	*Domain 3. Mechanisms of change:* *how NBHIs are thought to produce change.*			
1	Participation in everyday life	6.3	100%	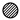
2	Social communities	6.6	92%	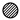
3	Nature experiences and nature interaction	6.3	84%	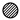
4	Physical activity	6.2	84%	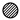
5	Body awareness	6.2	77%	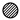
6	Coping ability/self-efficacy	6.2	77%	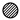
7	Sensory experiences	5.9	77%	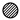
8	Exchange of experiences	5.8	69%	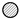
9	Self-reflection	5.6	69%	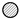
10	Empowerment	5.9	61%	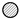

Agreement was defined as the proportion of respondents selecting categories 5–7 (somewhat agree to strongly agree) on a 7-point Likert scale. The table presents expert agreement across five domains central to the development and delivery of NBHIs. Consensus was defined a priori as ≥70% agreement. To aid interpretation, colour coding is used: 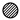 consensus (≥70%), 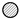 near consensus (60–69%), 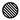 no consensus (<60%).

**Table 3:** Comparative Ranking of Mechanisms of Change Across Delphi Rounds.

Rank Round 1	Rank Round 2	Mechanism of Change (Round 2)
**3**	**1 ↑**	**Nature experiences and nature interaction**
**2**	**2 →**	**Social communities**
**4**	**3 ↑**	**Physical activity**
**-**	**4 New**	**Mindfulness/presence**
**7**	**5 ↑**	**Sensory experiences**
**5**	**6 ↓**	**Body awareness**
**8**	**7 ↑**	**Exchange of experiences**
**10**	**8 ↑**	**Empowerment**
**9**	**9 →**	**Self-reflection**
**6**	**10 ↓**	**Coping ability/self-efficacy**
**1**	**11 ↓**	**Participation in everyday life**

The table presents a comparison of mean ranks assigned by respondents in Round 2 with the relative position of each mechanism in Round 1. Lower mean values in Round 2 indicate higher perceived relevance (1 = most relevant). The table illustrates how prioritisation shifted between rounds. Arrows indicate changes in ranking between Delphi rounds: ↑ indicates an increase in ranking in Round 2, → indicates no change in ranking, and ↓ indicates a decrease in ranking in Round 2.

**Table 4:** Prioritisation of Outcomes for Evaluating NBHIs.

Rank	Outcome Measure	Mean Score
**1**	Mental well-being	1.4
**2**	Quality of life	2.7
**3**	Functioning	2.8
**4**	Symptom reduction	3.2

## Data Availability

The data presented in this study may be available on request from the corresponding author due to privacy of the respondents.

## Abbreviations

The following abbreviations are used in this manuscript:

NBHI	Nature-based health intervention
MRC	Medical Research Counsil

## Appendix A. Items, Ranking and Open-Ended Questions Delphi Round 1

**Item **	**Question**	**Preparatory Sources Informing Delphi Items**	
	**Domain 1. Target group**	**Systematic review**	**Stakeholder dialogues**
1	*To what extent do you agree that the target group benefits most from participating in a diagnosis-specific group intervention (*e.g., *only individuals with stress or anxiety)?*	Systematic literature review	Theme: *Needs of the Target Group*
2	*To what extent do you agree that the target group benefits most from participating in a mixed group intervention involving anxiety, depression and/or stress?*	Systematic literature review	Theme: *Needs of the Target Group*
	*Open-ended question: Please elaborate your considerations here:*		
	**Domain 2. Professionals**		
3	*To what extent do you agree that NBHIs should be delivered by an interdisciplinary team? (*e.g., *a physiotherapist and a nurse, or a psychologist and a nature guide)*		Theme: *Competency Development*
	*To what extent do you agree that the following professional groups should deliver the nature-based health interventions to the target group?*		Theme: *Competency Development*
4	- Nurse		
5	- Medical doctor		
6	- Physiotherapist		
7	- Occupational therapist		
8	- Psychologist		
9	- Nature educator		
10	*To what extent do you agree that the professional’s knowledge and experience with NBHIs is essential?*		Theme: *Competency Development*
11	*To what extent do you agree that the professional’s knowledge and experience with the target group—people with mild to moderate anxiety, depression and/or stress—is essential?*		Theme: *Competency Development*
	Open-ended question: *Are there any other important professional groups not mentioned here?*		
	**Domain 3. Mechanisms of change**		
	*Based on your knowledge of the target group, to what extent do you agree that the following mechanisms of change should be integrated into a NBHI for people with mild to moderate anxiety, depression and/or stress?*		Themes: *Added Value of Nature* AND *Needs of the Target Group*
12	- Nature experiences and nature interaction		
13	- Exchange of experiences		
14	- Sensory experiences		
15	- Social communities		
16	- Physical activity		
17	- Empowerment		
18	- Body awareness		
19	- Self-reflection		
20	- Coping ability/self-efficacy		
21	- Participation in everyday life		
	Open-ended question: *Are there any other important mechanisms of change you think should be included?*		
	**Domain 4. Outcome selection**		
	*We are interested in your assessment of which outcomes are most important to measure. How would you rank the following four outcomes? (1 being the one you consider most important to measure)*	Systematic literature review	Theme: *Measuring the Intervention*
	- Symptoms (fx. DASS-21)		
	- Quality of life (fx. EQ-5D)		
	- Well being (fx. WHO-5)		
	- Functioning (fx PSFS)		
	Open-ended question: *Are there any other more important outcomes not mentioned here?*		

## Appendix B. Ranking and Open-Ended Questions Delphi Round 2

**Question**
**Domain 1. Target group**
In the first round of the Delphi survey, we asked whether the target group benefits more from participating in a diagnosis-specific group program (e.g., only stress or anxiety) or in a group program across diagnoses such as anxiety, depression and/or stress. As shown in the table—with an average score of 4.1 and 4.4 respectively—consensus was not achieved, which requires a score of 5.0 or higher. We recognize that both options have certain advantages and disadvantages, which makes the question complex. We are interested in hearing your reflections and perspectives on the pros and cons of choosing one type of group composition over the other. Please elaborate your considerations here:
**Domain 2. Professionals**
In the first round of the Delphi survey, we asked whether NBHIs should be carried out by an interdisciplinary group (e.g., a physiotherapist and nurse, or a psychologist and a nature guide). As shown in the table, near consensus was achieved—with an average of 5.6 and 69%—greeing that an interdisciplinary team should provide the NBHI. If you have additional comments, you are very welcome to elaborate here:
In the first round of the Delphi survey, we asked which professionals should deliver the NBHIs for the target group. As shown in the table, we achieved consensus (>5.0) that physiotherapists, nature educators and psychologists are relevant to involve as the primary professionals in delivery of NBHIs for the target group—with nurses, medical doctors and occupational therapists reaching near consensus. If you have additional comments, you are very welcome to elaborate here:
In the first round of the Delphi survey, we asked about the competencies of the professionals, aiming to determine the weighting between knowledge and experience with NBHIs and with the target group. As shown in the table, there is consensus (>5.0) that both aspects should be weighted equally in the education and selection of professionals. If you have additional comments, you are very welcome to elaborate here:
**Domain 3. Mechanisms of change**
In this round, we ask you to rank the mechanisms of change in order of priority. How would you rank the following mechanisms of change? (1 is the one you find most relevant)
- Nature experiences and nature interaction
- Exchange of experiences
- Sensory experiences
- Social communities
- Physical activity
- Empowerment
- Body awareness
- Self-reflection
- Coping ability/self-efficacy
- Participation in everyday life
- Being present/ mindfulness (new)
Open-ended question: If you have additional comments, you are very welcome to share them here:
Open-ended question: Are there any unintended consequences we should be particularly aware of in relation to NBHIs for the target group?
**Domain 4. Outcome selection**
In *Nature Impact*, we investigate which available measurement tools can help evaluate the effect of NBHIs on mental health in people with mild to moderate anxiety, depression and/or stress. As part of this work, we are developing a questionnaire composed of several core instruments. In the project’s first phase, it was determined that nature connectedness should be included as the first outcome. In the first Delphi round, we asked you to rank outcomes (well-being, quality of life, functioning and symptoms) based on their relevance. As shown in the table, there is broad agreement that well-being should be included. Based on this, we chose to omit quality of life as an outcome, since several participants noted a potential overlap. In this round of the Delphi survey, we would like your input on prioritising between symptoms and functioning as the final outcome to be included in the questionnaire. Symptoms scored relatively low, which surprised us and made us discuss its relevance to the *Nature Impact* research project. On one hand, measuring symptoms is widely used in healthcare and may be important for implementing the intervention and for the impact of the results. On the other hand, using symptoms as a measurement may maintain a less contemporary understanding and approach to mental health. Regarding functioning and the measurement tools available, we see the challenge that the activities in NBHIs are not directly linked to what is typically measured. We would like your perspectives on this discussion, as well as your considerations about whether functioning or symptoms should be included in a questionnaire along with nature connectedness and well-being. Please elaborate your considerations here:
If you have additional comments or points of attention, you are very welcome to share them here:
